# Rhodobacter capsulatus PG Lipopolysaccharide Blocks the Effects of a Lipoteichoic Acid, a Toll-Like Receptor 2 Agonist

**DOI:** 10.32607/actanaturae.11747

**Published:** 2022

**Authors:** S. V. Zubova, N. I. Kosyakova, S. V. Grachev, I. R. Prokhorenko

**Affiliations:** Institute of Basic Biological Problems of RAS FRC PSCBR RAS, Pushchino, 142290 Russia; Clinical Hospital at the Pushchino Research Center, Pushchino, 142290 Russia; First Moscow State Medical University named I.M. Sechenov of Russia Health Ministry (Sechenov University), Moscow, 119991 Russia

**Keywords:** lipopolysaccharide, Rhodobacter capsulatus, lipoteichoic acid, TLR, CD14, cytokines

## Abstract

Lipopolysaccharides (LPS) and lipoteichoic acids (LTA) are the major inducers of the inflammatory response of blood cells caused by Gram-negative and some Gram-positive bacteria. CD14 is a common receptor for LPS and LTA that transfers the ligands to TLR4 and TLR2, respectively. In this work, we have demonstrated that the non-toxic LPS from Rhodobacter capsulatus PG blocks the synthesis of pro-inflammatory cytokines during the activation of blood cells by Streptococcus pyogenes LTA through binding to the CD14 receptor, resulting in the signal transduction to TLR2/TLR6 being blocked. The LPS from Rhodobacter capsulatus PG can be considered a prototype for developing preparations to protect blood cells against the LTA of gram-positive bacteria.

## INTRODUCTION

Studying the mechanisms of inflammation induced by ligands of differing nature is one of the priorities in modern biomedicine. This work considers the possibility of using lipopolysaccharide (LPS) from Rhodobacter capsulatus PG, a non-toxic endotoxin antagonist, to study the mechanisms underwriting the functional responses of innate immunity cells to pathogen-associated molecular patterns (PAMP) of differing nature. LPS and lipoteichoic acids (LTA), the central elements of the cell wall of Gram-negative and Gram-positive bacteria, exhibit immunostimulatory activity. LPS are glycolipids with three structural domains: lipid A, core oligosaccharide, and O-antigen, and they are localized in the outer membrane of Gram-negative bacteria. LTA are amphiphilic di- and triacylated lipopeptides anchored on the outer side of the cytoplasmic membrane of Gram-positive bacteria. In some aspects, LTA can be considered the equivalent of LPS, which is responsible for the development of the septic shock induced by Gram-positive bacteria [1]. TLR4 and TLR2 when expressed on the surface of blood cells can recognize these biologically active molecules. TLR4 has been identified as a specific receptor for LPS, inducing the release of pro-inflammatory cytokines by monocytes and macrophages stimulated by endotoxins [2]. TLR2 recognizes the di- or triacylated LTA of Gram-positive bacteria by triggering the immune response [3, 4]. The LTA from Streptococcus pyogenes, Staphylococcus aureus, and Streptococcus pneumonia bind directly to TLR2 [5, 6, 7]. The blood LBP protein, which binds to LPS and transfers it as a monomer to the membrane-bound receptor CD14, then to MD-2 and TLR4, is involved in the delivery of LPS to the receptor [8]. LBP and CD14 are also involved in LTA delivery to TLR2 [4]. CD14 constitutes part of the multi-ligand receptor complex, mediating a variety of cellular responses related to signal transduction from TLR2 and TLR4 [9]. CD14 enhances the TLR2 activation by facilitating lipopeptide binding and TLR2 heterodimerization with TLR1 or TLR6. The activation of the TLR2/ TLR6 complex by diacylated lipopeptides, particularly LTA, involves the CD36 receptor [10]. For TLR4 to function as an LPS receptor, the myeloid differentiation factor MD-2 is required [11]. MD-2 is physically associated with TLR2 but weaker than it is with TLR4 [12]. This accessory molecule has been shown to enhance the TLR2-mediated responses to LTA [13]. Unlike TLR4, which transmits signals as a homodimer (TLR4)2 when responding to LPS, TLR2 forms a heterodimer with TLR6 or TLR1 when recognized by LTA [14, 15]. The cell wall bacterial components LTA and LPS trigger the intracellular signaling cascade through TLR2 and TLR4 via a similar signaling pathway, that activates the transcription factors NF- κB, PKC, PI3K, ERK, JNK, and p38 MAPK and synthesizes the pro-inflammatory cytokines TNF-α, IL- 1β, IL-6 and chemokine IL-8 [16]. LPS from a wide range of non-enterobacterial bacteria activate the myeloid cell line via TLR2 [17, 18]. The features of the lipid A of these LPS include a presence of phosphorylated diglucosamine, the length of hydrocarbon chains of fatty acid residues different from the chain length of enterobacterial LPS, or branched acyl chains [19]. The non-toxic LPS of the Gram-negative phototrophic bacterium Rhodobacter capsulatus PG functions as an endotoxin antagonist [20, 21]. This LPS can block blood cell activation, resulting in a wide range of pro-inflammatory cytokines being released caused by endotoxins [22]. E5531, a synthetic analog of lipid A from R. capsulatus, suppresses TNF-α production by human blood monocytes activated by E. coli LPS 0111:B4 or Staphylococcis faecalis LTA, exhibiting almost no activity of its own [23].

The structure of the non-toxic lipid A of the LPS from Rhodobacter capsulatus includes diphosphorylethanolamine at C-1, phosphorylethanolamine at C-4’, and an unsaturated fatty acid (12:1) in the disaccharide backbone [24]. These structural features of lipid A allowed us to hypothesize that Rhodobacter capsulatus PG LPS, similar to E5531, could compete with S. pyogenes LTA for TLR2 by blocking the activation of pro-inflammatory cytokine synthesis by blood cells.

## EXPERIMENTAL

The research was performed on the whole blood of healthy volunteers of both sexes, with ages ranging from 25 to 30 years. All subjects gave written consent to participate in the study. The study protocol complies with the Declaration of Helsinki of the World Medical Association (2013) and was approved by the Local Ethics Committee of the Hospital of the Pushchino Scientific Center (No. 2 of April 10, 2014). Peripheral blood was collected under clinical conditions using vacutainers (Becton Dickinson and Company, United Kingdom) treated with sodium heparin (17 units/ml).

**Activation of blood cells by LPS and LTA **We studied the effect of LPS and LTA on cytokine and chemokine synthesis by diluting blood in RPMI 1640 medium at a ratio of 1 : 10 and incubating with E. coli LPS 055:B5 (100 ng/ml), S. enterica serotype Typhimurium LPS (100 ng/ml), S. pyogenes LTA (1000 ng/mL) (Sigma-Aldrich, USA), or Rhodobacter capsulatus PG LPS (1000 ng/mL) in various combinations for 6 and 24 h at 37°C in 5% CO_2_. The Rhodobacter capsulatus PG LPS was obtained according to the method described previously [25]. We determined the antagonistic effect of Rhodobacter capsulatus PG LPS against E. coli LPS, S. enterica LPS, or S. pyogenes LTA in various combinations by preincubating blood with Rhodobacter capsulatus PG LPS for 30 min, followed by the addition of LPS or LTA. To determine the role of the CD14 receptor in cell activation, we preincubated the blood with antibodies (Ab) to CD14 (2 μg/ml) (Purified Anti-human CD14 Clone M5E2, BioLegend, USA) for 30 min at 4°C and then added LPS or LTA. The samples were incubated for 6 and 24 h at 37°C in 5% CO_2_. Once incubated, the blood cells were precipitated by centrifugation (300 g, 10 min). The supernatants were collected and stored at −20°C until the cytokine and chemokine contents were determined.

**Cytokine and chemokine content **The content of cytokines and chemokines was determined using TNF-α, IL-6, IL-1β, and IL-8 ELISA kits (Vector-BEST, Russia) according to the manufacturer’s protocol. The optical density of the samples was determined using a STAT FAX 3200 ELISA analyzer (Awareness Technology Inc., USA) at a wavelength of 450 nm.

**Statistical analysis **Statistical processing and graphical representation of the results were performed using nonparametric statistics in Origin Pro 7.5 and Microsoft Office Excel 2010 (AtteStat plugin). The results were presented as values with upper and lower quartiles (IQR). The statistical significance of the differences between median values was determined by the Mann-Whitney test (p < 0.05).

## RESULTS

**Fig. 1:**
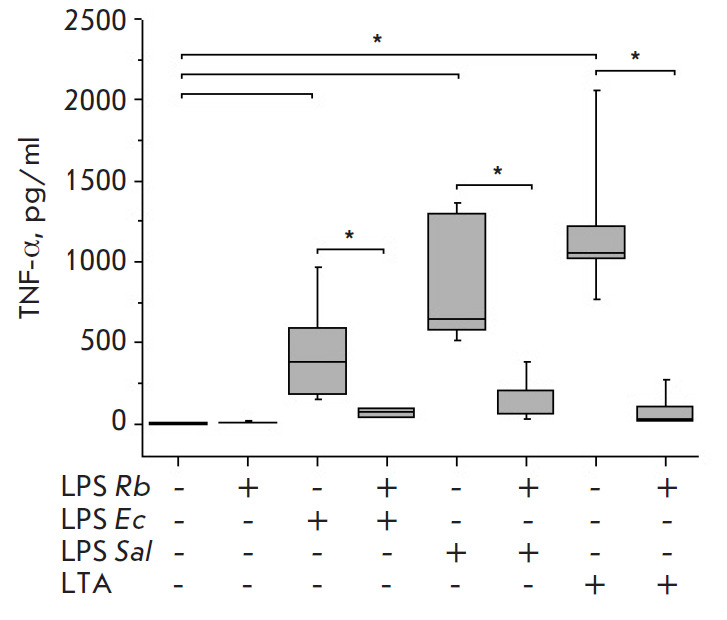
Effect of *R. capsulatus *PG LPS on TNF-α secretion upon activation of blood cells by *E. coli *LPS, *S. enterica *LPS, or *S. piogenes *LTA, *n *= 7. *p < 0.05

**Fig. 2:**
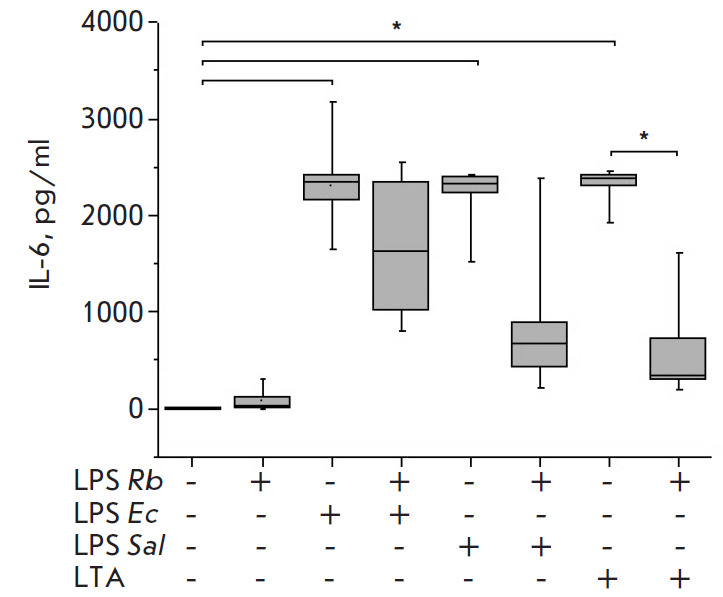
Effect of *R. capsulatus *PG LPS on IL-6 secretion upon activation of blood cells by *E. coli *LPS, *S. enterica *LPS, or *S. piogenes *LTA, *n *= 7. *p < 0.05

**Fig. 3:**
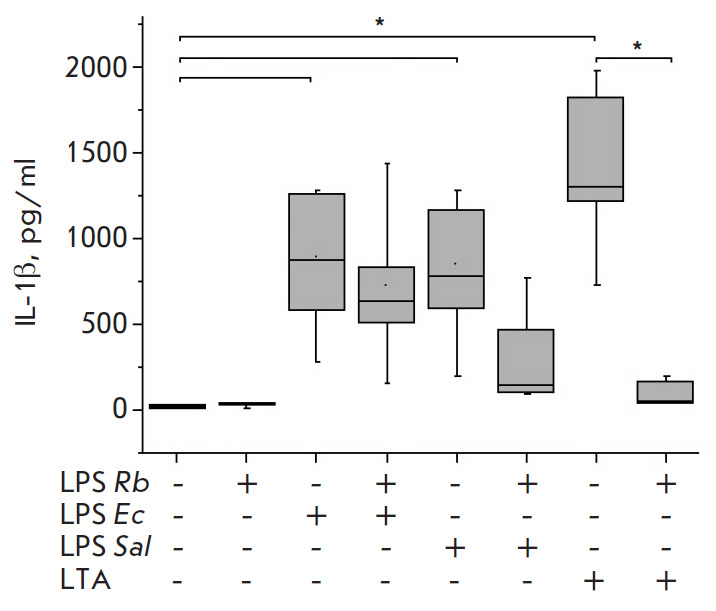
Effect of *R. capsulatus *PG LPS on IL-1β secretion upon activation of blood cells by *E. coli *LPS, *S. enterica *LPS, or *S. piogenes *LTA, *n *= 7. *p < 0.05

**Fig. 4:**
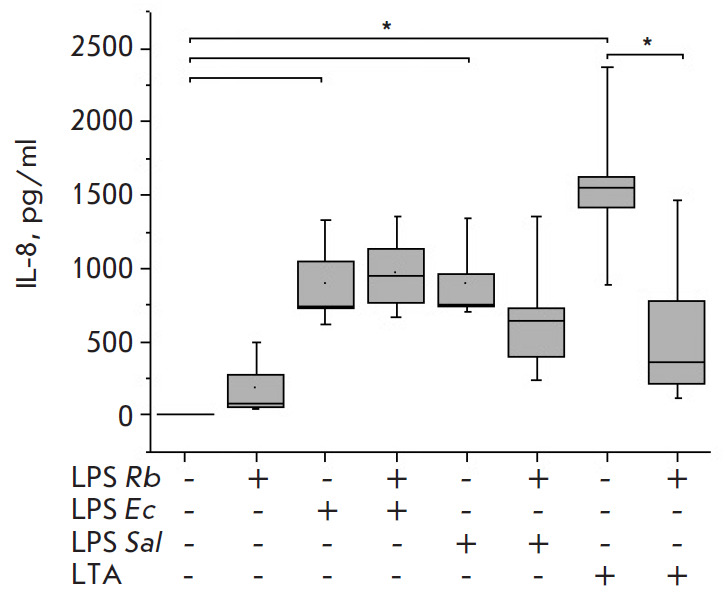
Effect of *R. capsulatus *PG LPS on IL-8 secretion upon activation of blood cells by *E. coli *LPS, *S. enterica *LPS, or *S. piogenes *LTA, *n *= 7. *p < 0.05

E. coli LPS or S. enterica LPS stimulated significant, similarly high, production of the pro-inflammatory cytokines TNF-α (Fig. 1), IL-6 (Fig. 2), and IL-1β (Fig. 3), as well as the inflammatory chemokine IL-8 (Fig. 4), whose production significantly exceeded control values. LTA activation also resulted in the production of high levels of the cytokines and chemokines analyzed. The level of synthesis of the later cytokine IL-1β and chemokine IL-8 in response to S. pyogenes LTA exceeded the levels when activated by E. coli LPS or S. enterica LPS (Fig. 3, Fig. 4).

Non-toxic Rhodobacter capsulatus PG LPS at a concentration 10-fold higher than that of the E. coli and S. enterica endotoxins and at equal concentration with S. pyogenes LTA did not stimulate the cells to produce TNF-α, IL-6, and IL-1β (Fig. 1-3). The amount of chemokine IL-8 in the blood in response to Rhodobacter capsulatus PG LPS slightly increased compared to the control but was significantly lower than that during the activation of blood cells by endotoxins or S. pyogenes LTA (Fig. 4). The study of the ability of Rhodobacter capsulatus PG LPS to protect blood cells from the action of the E. coli and S. enterica endotoxins revealed that the Rhodobacter capsulatus PG LPS suppressed the synthesis of the TNF-α, IL-6, and IL-1β cytokines in the blood, with the blocking response to S. enterica LPS being stronger than that to E. coli LPS (Fig. 1–3).

No protective effect of Rhodobacter capsulatus PG LPS against the endotoxins was observed according to the IL-8 chemokine synthesis (Fig. 4). IL-8 is an important mediator of the host response to inflammation and infection [26]. It is assumed that the cell response to an exposure to bacterial agents and IL-8 synthesis is induced earlier than the IL-6 synthesis [27].

Upon the activation of the cells with S. pyogenes LTA, pre-incubation of blood with Rhodobacter capsulatus PG LPS resulted in a significant decrease in the synthesis of the pro-inflammatory cytokines TNF-α, IL-6 and IL-1β and chemokine IL-8 (Fig. 1–4). The data obtained suggest that the LPS from Rhodobacter capsulatus PG exhibit antagonistic activity not only against endotoxins, but also against the S. pyogenes LTA.

**Fig. 5:**
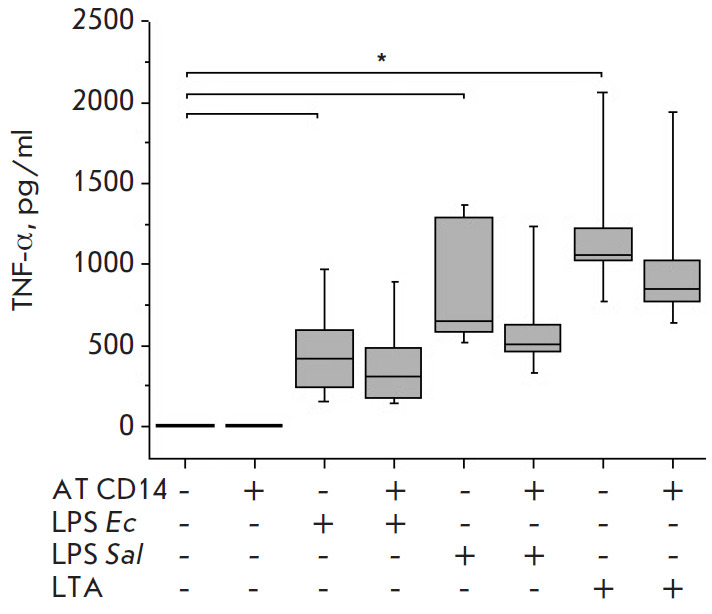
Effect of Ab CD14 on TNF-α secretion upon activation of blood cells by *E. coli *LPS, *S. enterica *LPS, *S. pyogenes *LTA, *n *= 7. *p < 0.05

In the control samples, Ab to CD14 did not affect the activation of the TNF-α synthesis in blood cells (Fig. 5). Pre-incubation of blood with Ab to CD14, followed by the activation of E. coli LPS, S. enterica LPS, or S. pyogenes LTA cells more markedly reduced the TNF-α synthesis induced by LTA than by endotoxins.

## DISCUSSION

Toll-like receptors (TLRs) activate the cells of the innate immune system by recognizing various microorganisms through pathogen-associated molecular patterns (PAMPs), particularly LPS of Gram-negative bacteria and LTA of Gram-positive bacteria. TLR4 receptors recognize LPS, the central inducers of the inflammatory responses induced by Gram-negative bacteria, and TLR2 recognizes LTA, the inducers of the inflammatory response triggered by Gram-positive bacteria [3]. Both receptors are capable of signaling by forming a homodimer (TLR4)2 or a TLR2/TLR6 heterodimer, respectively. Variations in the number of acyl chains in endotoxin lipid A can attenuate signaling through TLR4 and alter the host’s immune response to the pathogen [28]. TLR4/MD-2 recognizes hexaacylated E. coli lipid A as an agonist. The structural changes in the lipid A of other Gram-negative bacteria reduce their activity in the receptor complex, compared to hexaacylated lipid A. When examining the ability of E5531, a pentaacylated synthetic analog of lipid A of Rhodobacter capsulatus, to inhibit the binding of E. coli LPS to human monocytes, was calculated the affinity of E5531 to the cells to be 24 times lower than that of E. coli LPS [23]. We used Rhodobacter capsulatus PG LPS in concentrations 10-fold higher than those of endotoxins to block the effects of E. coli LPS or S. enterica LPS. The LPS of Rhodobacter capsulatus PG was found to block the synthesis of the pro-inflammatory cytokines TNF-α, IL-6, and IL-1β in the cells activated by S. enterica LPS stronger than E. coli LPS. The antagonistic activity of the LPS of Rhodobacter capsulatus PG against the S. pyogenes LTA was significantly stronger when equal weight concentrations of Rhodobacter capsulatus PG LPS and S. pyogenes LTA were used. The ability of Rhodobacter capsulatus PG LPS to protect the cells from activation cytokine synthesis by agonists was reduced in the series of S. pyogenes LTA > S. enterica LPS > E. coli LPS (Fig. 1–3). The CD14 receptor, involved not only in ligand recognition by the TLR4 and TLR2 receptors, but also in the activation of cytokine synthesis by the cells, plays a critical role in both LPS and LTA signal transduction [6, 29]. The CD14 receptors expressed on the cell surface bind with high affinity to the molecular ligands associated with various pathogens. Subsequently, CD14 transmits LPS to the TLR4/ MD-2 signaling complex [30]; and LTA, to the TLR2/ TLR6 complex [4]. CD14 and CD36 act as TLR2 co-receptors in the monocyte response to LTA. Blocking these receptors with antibodies inhibits the LTA-induced release of TNF-α by monocytes, indicating the involvement of these receptors in LTA binding to the plasma membrane and NF-κB activation [31]. On human monocytes, Streptococcus sanguis LTA has been shown to compete with Salmonella abortusequi LPS for binding to CD14. However, the LPS binding to CD14 has been found to be completely inhibited if the LTA concentration is 100-fold higher than the LPS concentration [32].

To validate this assumption and understand the mechanism of suppression of cell activation by Rhodobacter capsulatus PG LPS, we blocked blood cell CD14 receptors using mAbs prior to activation by the LPS or LTA agonist. The low percentage of activation reduction observed (compared to the data in [23]) upon blocking of the CD14 receptors is obviously related to the specificity of the antibodies we used (Clone M5E2). The pre-incubation of blood with Ab CD14 before the activation of the cells by E. coli LPS, S. enterica LPS, or S. pyogenes LTA more markedly reduced the TNF-α synthesis induced by LTA than by the endotoxins. The results obtained demonstrate that CD14 is involved in the activation and signal transduction to cytokine synthesis from LPS and LTA, with this involvement decreasing in the series of S. pyogenes LTA > S. enterica LPS > E. coli LPS (Fig. 5), similar to the decreasing efficiency of Rhodobacter capsulatus PG LPS protection from cell activation by the agonists used (Fig. 1-3).

Two possible mechanisms for blocking cell activation by Rhodobacter capsulatus PG LPS can be suppose here. They are related to the different affinities of the studied ligands for the CD14 receptors: blocking at the level of interaction with the CD14 receptor or at the level of activation of the TLR4/MD-2 or TLR2/TLR6 receptor complex.

## CONCLUSION

Our results have revealed that the non-toxic LPS of Rhodobacter capsulatus PG blocks the synthesis of pro-inflammatory cytokines upon blood cell activation by S. pyogenes LTA through binding to the CD14 receptor, resulting in a suppression of signal transduction to TLR2/TLR6. To conclude, we believe that the LPS of Rhodobacter capsulatus PG can be considered a prototype for developing preparations to protect blood cells from the action of LTA of Gram-positive bacteria.

## Abbreviations

CD: cluster of differentiation
ERK: extracellular signal-related kinase
IL: interleukin
JNK: c-Jun N-terminal kinase
LBP: LPS-binding protein
LPS: lipopolysaccharide
LTA: lipoteichoic acid
MAPK: mitogen-activated protein kinase
MD-2: myeloid differentiation protein 2
NF-κB: nuclear factor kappa B
PAMP: pathogen-associated molecular patterns
PI3K: phosphatidylinositol 3 kinase
PKC: protein kinase C
TLR: Toll-like receptor
TNF-α: tumor necrosis factor α

## References

[1] Ginsburg L. (2002). Lancet. Infect. Dis..

[2] Zhang G., Meredith T.C., Kahne D. (2013). Curr. Opin. Microbiol..

[3] Schwandner R., Dziarski R., Wesche H., Rothe M., Kirschning C.J. (1999). J. Biol. Chem..

[4] Schroder N.W.J., Morath S., Alexander C., Hamann L., Hartung T., Zahringer U., Gobel U.B., Weber J.R., Schumann R.R. (2003). Biol. Chem..

[5] Im J., Choi H.S., Kim S.K., Woo S.S., Ryu Y.H., Kang S.S., Yun C.H., Han S.H. (2009). Cancer Lett..

[6] Kang J.Y., Nan X., Jin M.S., Youn S.J., Ryu Y.H., Mah S., Han S.H., Lee H., Paik S.G., Lee J.O. (2009). Immunity..

[7] Fieber C., Janos M., Koestler T., Gratz N., Li X-D., Castiglia V., Aberle M., Sauert M., Wegner M., Alexopoulou L. (2015). PLoS One..

[8] Ryu J.-K., Kim S.J., Rah S.-H., Kang J.I., Jung H.E., Lee D., Lee H.K., Lee J.-O., Park B.S., Yoon T.-Y., Kim H.M. (2017). Immunity. 2017. V..

[9] Schmitz G., Orso E. (2002). Curr. Opin. Lipidol..

[10] Triantafilou M., Gamper F.G., Haston R.M., Mouratis M.A., Morath S., Hartung T., Triantafilou K. (2006). Biol. Chem..

[11] Shimazu R., Akashi S., Ogata H., Nagai Y., Fukudome K., Miyake K., Kimoto M. (1999). J. Exp. Med..

[12] Dziarski R., Wang Q., Miyake K., Kirsching C.J., Gupta D.J. (2001). J. Immunol..

[13] Dziarski R., Gupta D.J. (2000). Endotox. Res..

[14] Ozinsky A., Underhill D.M., Fontenot J.D., Hajjar A.M., Smith K.D., Wilson C.B., Schroeder L., Aderem A. (2000). Proc. Natl. Acad. Sci. USA..

[15] Henneke P., Morath S., Uematsu S., Weichert S., Pfitzenmaier M., Takeuchi O., Müller A., Poyart C., Akira S., Berner R. (2005). Immunol..

[16] Su S.-H., Hua K.-F., Lee H., Chao L.K., Tan S.-K., Lee H., Yang S.-F., Hsu H.-Y. (2006). Clin. Chim. Acta..

[17] Yokota S., Ohnishi T., Muroi M., Tanamoto K., Fujii N., Amano K. (2007). FEMS Immunol. Med. Microbiol..

[18] Girard R., Pedron T., Uematsu S., Balloy V., Chignard M., Akira S., Chaby R. (2002). J. Cell Sci..

[19] Erridge C., Pridmore A., Eley A., Stewart J., Poxton I.R. (2004). J. Med. Microbiol..

[20] Katzke N., Bergmann R., Jaeger K.-E., Drepper T. (2012). Methods Mol. Biol..

[21] Prokhorenko I.R., Grachev S.V., Zubova S.V. (2010). Patent for invention RU № 2392309 of 20.06.2010..

[22] Kabanov D.S., Serov D.A., Zubova S.V., Grachev S.V., Prokhorenko I.R. (2016). Biochemistry (Moscow)..

[23] Kawata T., Bristol J.R., Rossignol D.P., Rose J.R., Kobayashi S., Yokohama H., Ishibashi A., Christ W.J., Katayama K., Yamatsu I., Kishi Y. (1999). Br. J. Pharmacology..

[24] Krauss J.H., Seydel U., Weckesser J., Mayer H. (1989). Eur. J. Biochem..

[25] Makhneva Z.K., Vishnevetskaya T.A., Prokhorenko I.R. (1996). Pric. Biochim. Microbe..

[26] Baggiolini M., Walz A., Kunkel S.L. (1989). J. Clin. Invest..

[27] Hirao Y., Kanda T., Aso Y., Mitsuhashi M., Kobayashi I. (2000). Lab. Med..

[28] Kawai T., Akira S. (2010). Nat. Immunol..

[29] Zanoni I., Ostuni R., Marek L.R., Baressi S., Barbalat R., Barton G.M., Granucci F., Kagan J.C. (2011). Cell..

[30] Wright S.D., Ramos R.A., Tobias P.S., Ulevitch R.J., Mathison J.C. (1990). Science..

[31] Nilsen N.J., Deininge S., Nonstad U., Skjeldal F., Husebye H., Rodionov D., von Aulock S., Hartung T., Lien E., Bakke O., Espevik T.J. (2008). Leukoc. Biol..

[32] Sugawara S., Arakaki R., Rikiishi H., Takada H. (1999). Infect. Immunol..

